# Changes in Psychological and Cognitive Outcomes after Green versus Suburban Walking: A Pilot Crossover Study

**DOI:** 10.3390/ijerph16162894

**Published:** 2019-08-13

**Authors:** Junia N. de Brito, Zachary C. Pope, Nathan R. Mitchell, Ingrid E. Schneider, Jean M. Larson, Teresa H. Horton, Mark A. Pereira

**Affiliations:** 1 Division of Epidemiology & Community Health, School of Public Health, University of Minnesota, 1300 S 2nd St, Suite 300 Minneapolis, MN 55455, USA; 2 Department of Forest Resources, University of Minnesota, 1530 Cleveland Ave North, Suite 301b St. Paul, MN 55108, USA; 3 Minnesota Landscape Arboretum, Earl E. Bakken Center for Spirituality & Healing, University of Minnesota, 3675 Arboretum Drive, Chaska, MN 55318, USA; 4 Department of Anthropology, Northwestern University, 1819 Hinman Avenue, Rm 302 Evanston, IL 60208, USA

**Keywords:** green exercise, physical activity, anxiety, mood, directed-attention

## Abstract

This study investigated the acute effects of repeated walking sessions within green and suburban environments on participants’ psychological (anxiety and mood) and cognitive (directed-attention) outcomes. Twenty-three middle-aged adults (19 female) participated in a non-randomized crossover study comprised of once-weekly 50-min moderate-intensity walking sessions. Participants walked for three weeks in each of two treatment conditions: green and suburban, separated by a two-week washout period. Eleven participants completed green walking first and 12 suburban walking first. For each walk, we used validated psychological questionnaires to measure pre- and post-walk scores for: (1) mood, evaluated via the Positive and Negative Affect Schedule (PANAS); (2) anxiety, assessed by the State-Trait Anxiety Inventory (STAI-S); and (3) directed-attention, measured using the visual Backwards Digit Span test (BDS). Repeated measures linear mixed models assessed pre- to post-walk changes within-treatment conditions and post-walk contrasts between-treatment conditions. Results indicated that anxiety decreased after green walking and increased after suburban walking (−1.8 vs. +1.1 units, respectively; *p* = 0.001). For mood, positive affect improved after green walking and decreased after suburban walking (+2.3 vs. −0.3 units, respectively; *p* = 0.004), and negative affect decreased after green walking and remained similar after suburban walking (−0.5 vs. 0 units, respectively; *p* = 0.06). Directed-attention did not improve from pre- to post-walk for either condition. Our results suggested that green walking may be more effective at reducing state anxiety and increasing positive affect compared to suburban walking.

## 1. Introduction

Physical activity (PA) has well-established health benefits—not only physiologically, but psychologically [1,2]. Literature indicates that regular PA participation is positively correlated with improvements in overall mental health, wellbeing, and mood, in addition to reductions in depression, anxiety, and stress symptomology [1,3,4]. Walking is the most popular form of moderate-intensity PA among adults [5], with this PA modality having not only wide-reaching physiological benefits [6], but also numerous psychological benefits. A review by Kelly and colleagues [7] noted walking’s robust ability to promote reduced anxiety and stress, while others highlighted walking’s ability to improve feelings of vigor [8] and positive affect (i.e., positive mood) [9]. However, a literature gap exists regarding how the walking setting impacts health outcomes [7].

Interaction with, and PA within nature may promote community health and wellbeing. In fact, the American Public Health Association (APHA) released a 2013 policy statement aimed at prioritizing access to nature-based areas and greenspace—emphasizing the need for safe walkable greenspaces across U.S. communities to promote active lifestyles across the lifespan [10]. Indeed, evidence has indicated that greenspace access increases the likelihood of walking participation and other forms of PA engagement, with the potential health benefits of “green exercise” (i.e., regular or repeated PA participation in nature-based green environments) noted [11,12]. Additionally, in 2017, the World Health Organization summarized key benefits of urban greenspace and stated that greenspace is an essential component for mental health and well-being [13]. Given the high prevalence of mental illness worldwide and the reduced greenspace accessibility in urban areas [13,14], research evaluating how access to greenspace can improve mental health and well-being has increased.

Empirical research has indeed suggested that exposure to, or exercise within natural environments improves several health outcomes in adults—physiologically and, of greater pertinence to our investigation, psychologically [15,16,17,18,19,20,21,22]. Among prior green exercise studies which have compared green exercise to other environments (e.g., indoor exercise), these investigations have suggested that not only does green exercise appear to promote greater physiological benefits in healthy adults (e.g., greater systolic blood pressure reductions), but may also lead to larger improvements in multiple psychological health outcomes (e.g., mood, well-being) [23,24,25].

Greater improvements in focus and directed-attention (i.e., cognitive ability to avoid being distracted by competing stimuli) [26] have also been reported after green exercise relative to indoor exercise [24]. Some theories have been proposed to support these findings and to explain how the natural environment affects cognitive capabilities. For instance, the Attention Restoration Theory states that nature-based interactions allow an individual to rest the neurocognitive inhibitory mechanism which filters out irrelevant stimuli when we are required to focus on specific tasks (e.g., work) [27]. This ‘neurocognitive rest period’ is posited to allow one to better adapt to various subsequent life stressors (e.g., work demands, family responsibilities) [27,28]. Additionally, the Stress Recovery Theory posits that exposure to restorative environments produces a more relaxed and positive emotional state thus fostering recovery from stress [29]. Beyond the benefits conferred by PA completed in other settings (e.g., indoors) and green exercise’s noted psychological benefits, green exercise may therefore possess restorative characteristics which improve cognition and enhance an individual’s ability to destress and subsequently adapt to life stressors [17]. Few studies, however, have concurrently examined the effect of repeated walking sessions conducted in a green environment (i.e., nature-based) relative to another outdoor environment on acute psychological (e.g., anxiety, mood) and cognitive outcomes (e.g., directed-attention) in healthy adults. Therefore, to better understand the role of walking in outdoor environments for enhanced psychological and cognitive outcomes, it is important to elucidate whether walking in outdoor green environments promotes greater psychological and cognitive benefits beyond that expected from walking in other outdoor environments where adults are likely to walk (e.g., suburban environments near their place of residence).

Other limitations of the current green exercise literature are important to note. First, most previous studies have employed cross-sectional designs investigating single exercise bouts in small samples, consisting, most frequently, of university students, thus limiting generalizability to other groups [30,31]. Given the emerging nature of the green exercise field, stronger experimental designs for assessing intervention efficacy are needed. A crossover study design offers advantages relative to traditional parallel trials when studying the efficacy of novel intervention methods (e.g., interaction effects of a physical activity intervention on environmental exposures). These advantages include the improved precision of study observations as each participant serves as his/her own control and the improved statistical power conferred by this design—even in smaller samples [32]. Second, previous studies have often compared green exercise to exercise performed in indoor settings [24,33,34]. A recent review noted that due to methodological issues and inconsistencies across studies, there is limited evidence to support the idea that green exercise promotes greater benefits relative to exercises performed in other environments (i.e., without the presence of nature) [35]. Third, and relatedly, few studies have compared green exercise to exercise completed in outdoor suburban or urban locations [36,37]. This latter point is important and aligns with the APHA’s 2013 greenspace conservation policy statement in addition to two reviews which have cited the need to study how regular walking completed in different settings might differentially influence psychological and cognitive health outcomes [7,38]. Therefore, rigorously comparing psychological and cognitive outcomes between repeated bouts of green walking and walking completed in outdoor suburban/urban locations is important and may have policy-level implications. 

In a sample of middle-aged adults, this study’s purpose was to investigate the acute pre- to post-effects of repeated walking sessions completed in a green environment (hereafter referred to as “green walking”) compared to walking in a suburban environment (hereafter referred to as “suburban walking”) on measures of (1) state anxiety; (2) mood (i.e., positive and negative affect); and (3) directed-attention. Using a crossover design, it was hypothesized that relative to repeated bouts of suburban walking, repeated bouts of green walking would demonstrate: (1) greater decreases in state anxiety scores; (2) larger improvements in mood scores (i.e., greater increases in positive affect and decreases in negative affect); and (3) greater improvements in directed-attention scores post-walk. 

## 2. Materials and Methods

### 2.1. Participants

Following ethical approval by the University of Minnesota Institutional Review Board (IRB) and ClinicalTrials.gov registration (NCT03442998), a convenience sample of 24 (20 females) healthy middle-aged adults (mean ± SD: 49.3 ± 6.7 years) was recruited, provided informed consent, and enrolled in our study. Data from 23 participants encompassed the final analytical sample (see details in the Results section). Participants were recruited via electronic advertisements and met the following inclusion criteria: (1) aged 35–59 years; (2) no contraindications to regular moderate-intensity walking; (3) no chronic disease diagnosis and not taking medication for any chronic disease (e.g., cardiovascular disease, diabetes, hypertension); (4) no antidepressant or anti-anxiety medication use; and (5) currently not exceeding PA guidelines (i.e., ≥150 min/week) [39]. Participants were compensated up to $200.00 US for study participation. Participant consent was obtained at the baseline visit, with all participant procedures performed in accordance with the ethical standards of the University IRB and with the 1964 Helsinki Declaration and its later amendments [40]. 

### 2.2. Study Design

**Figure 1:**
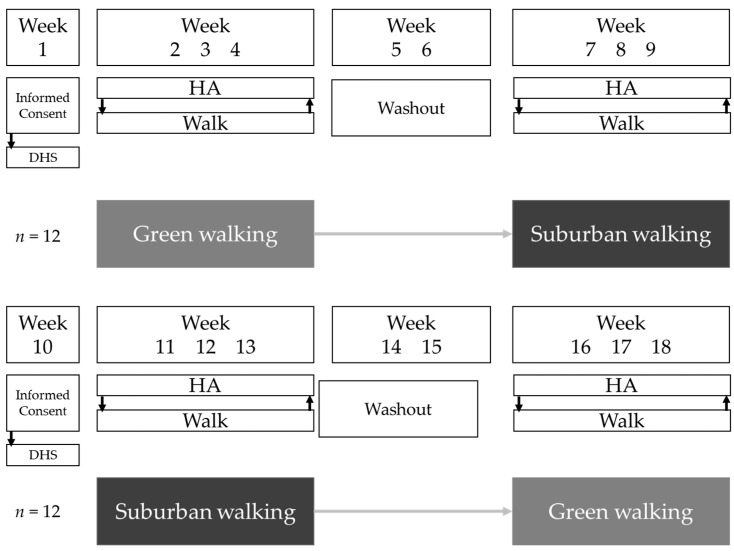
Study design. Abbreviations: demographic and health survey, DHS; health assessment, HA. HA refers to the assessment of psychological (State-Trait Anxiety Inventory (STAI-S) and Positive and Negative Affect Schedule (PANAS)) and cognitive outcomes (Backwards Digit Span test, BDS) measures administered to participants before and after each of the weekly 50-min walking sessions completed within the green and suburban settings.

The present study was a 9-week crossover trial during which all participants were exposed to two treatment conditions: (1) green walking and (2) suburban walking. Participants visited each location (i.e., green and suburban) once-weekly for three consecutive weeks, on the same day and time, with a two-week washout period in between treatment conditions (Figure 1). Due to limited study resources, simultaneous participant randomization to the two conditions was not possible. Therefore, the first 12 participants recruited were assigned to the following treatment sequence: green walking then suburban walking; the next 12 participants recruited were assigned the opposite treatment sequence: suburban walking then green walking. Regardless of treatment sequence, participants walked in both treatment conditions.

### 2.3. Environmental Settings

Green walking took place on the Wood Duck Trail of the Minnesota Landscape Arboretum (MLA), located 40 kilometers southwest from Minneapolis, MN. This unpaved trail features secluded areas surrounded by large trees as well as open views of grassland areas and a pond. The MLA is part of the University of Minnesota College of Food, Agricultural and Natural Resource Science and has more than 1200 acres of gardens and prairies in addition to several kilometers of trails. We chose the MLA because of the richness in vegetation and greenness. Suburban walks occurred approximately three kilometers away from the MLA’s trail on paved sidewalks adjacent to medium traffic roads located within a medium-density residential development area (town population of ~27,000 residents). Importantly, the trail and sidewalks had minimal inclination.

### 2.4. Measures

#### 2.4.1. Demographics

At each participant’s baseline visit, a demographic questionnaire was used to collect date of birth, sex, household income, employment status, and level of education. Additionally, participants’ weight and body fat percentage were assessed to the nearest 0.1 kilogram and 0.1%, respectively, using a calibrated electronic Tanita TBF-300A Body Composition Analyzer scale (Tanita Corp., Tokyo, Japan). Height was assessed to the nearest 0.1 centimeter using a Seca stadiometer (model 437; Seca, Hamburg, Germany). 

#### 2.4.2. Outcome Measures

Psychometrically-validated questionnaires were administrated individually to participants before and after each walk using an electronic tablet device. Prior to administering the questionnaires, research assistants loaded unique participant identifiers into an online Qualtrics survey portal (Qualtrics Inc.; Provo, UT) which stored participants’ questionnaire responses—allowing for later data download and analysis. Each questionnaire (reviewed below) was accompanied by detailed instructions, with a research assistant available to answer participants’ additional questions. Combined, these measures took approximately 15 min to complete.

State anxiety. The 20-item state anxiety subscale of the State-Trait Anxiety Inventory (STAI) assessed participant’s anxiety [41]. Participants were questioned about current anxiety-related feelings such as “I am tense; I am worried” and “I feel comfortable; I am relaxed” and responded on a 4-point Likert-type scale (1: not at all; 4: frequently so). The items on the subscale were summed, with higher scores indicating greater state anxiety (subscale score range: 10 to 40). Notably, the STAI possesses good internal consistency (Cronbach’s α = 0.86–0.95) and adequate test-retest reliability (ICCs = 0.65–0.75) [41].

Mood. Mood assessments were completed via the 20-item Positive and Negative Affect Schedule (PANAS) [42]. This measure presented participants with 20 different adjectives (e.g., interested, upset), with ten positive and ten negative affect descriptors comprising the positive affect and negative affect subscales, respectively. Using a 5-point Likert-type scale (1: very slightly or not at all; 5: extremely), the PANAS assessed to what extent the participant currently felt the listed positive or negative emotion. Scores for the positive and negative affect scales were summed (range 10–50), with higher scores on both subscales representing higher positive and negative mood, respectively. Watson et al. [42] reported good internal consistency for the positive affect (Cronbach α = 0.86–0.90) and the negative affect (Cronbach α = 0.84–0.87) subscales. Similarly, test-retest reliability correlations have been documented as adequate for the positive affect (ICCs = 0.47–0.68) and the negative affect (ICCs = 0.39–0.71) subscales [42].

Directed-attention. Directed-attention was assessed by the visual Backward Digit Span task (BDS) [43]. The BDS required participants to view a random sequence of numbers and, upon these numbers’ disappearance, type these numbers back into the tablet in a backwards sequence from what they initially appeared. The first sequence seen contained three numbers, and sequence length increased up to nine numbers, with two sequences of each length (i.e., 14 sequences total). For each correctly typed backwards sequence, participants received one point. Scores on the BDS task range from 0–14, with larger scores indicating greater attentional capacity.

#### 2.4.3. Procedures

Upon arrival at each walking location, participants were given a tablet to first complete the visual BDS task, after which the two psychological questionnaires were completed in the following order: (1) STAI and (2) PANAS. Once participants finished all questionnaires, participants were handed a stopwatch to time their walks as the use of any smartphones and/or smart device (e.g., smartwatch) was prohibited until the end of each visit. Participants were scheduled in a staggered manner that ensured they were walking alone and uninterrupted throughout the 50-min walk. This format also allowed participants to be observant of their environment (e.g., wildlife, road traffic), without the presence of another participant confounding study observations. We asked participants to stop only if needed (e.g., at road intersections, to tie an untied shoe) and although walking intensity was participant-determined, jogging and/or running were discouraged. Upon returning from each walk, participants were asked once again to complete the visual BDS task and the psychological questionnaires in the same order as before the walk. Following three weeks of once-weekly walking in the first condition (i.e., either green or suburban), participants had a two-week washout period before initiating three weeks of once-weekly walking in the second condition. Again, we administered all psychological and cognitive assessments to participants before and after each walking session in each condition to ensure our ability to assess acute changes in state anxiety, mood and directed-attention.

#### 2.4.4. Statistical Analysis

Prior to inferential data analyses, we first inspected all data for potential data recording/entry errors. After calculating scores for each psychological and cognitive outcome per the questionnaire-specific scoring methods, we performed normality testing—examining these data visually as well as via skewness/kurtosis values and associated Shapiro–Wilk statistics. 

For the main analyses, a repeated measures linear mixed models approach using PROC MIXED in SAS version 9.4 (SAS Institute Inc., Cary, NC, USA) was used to analyze the data from this crossover study. All pre- and post-walk data from each of the three consecutive weeks by condition (i.e., either green or suburban) were used in the analyses. We modeled separately repeated pre- and post-walk scores for state anxiety, mood, and directed-attention as well as repeated pre- to post-walk score changes as dependent variables. Treatment, period, and sequence were modeled as independent variables. We entered participant, treatment (green vs. suburban), sequence (green then suburban vs. suburban then green), time (walk week), and period into the models as categorical fixed variables. Period was included in the models because we did not randomize participants into a treatment sequence [44]. Analyses of the visual BDS scores were adjusted for participants’ baseline visual BDS score to control for the influence of any learned effect from the repeated administration of the visual BDS task. We nested participants within sequence as a random effect to control for variation in psychological outcomes scores between participants within different treatment sequences. A compound-symmetry structure was chosen to model correlations between repeated measurements over time as it demonstrated the smallest Akaike information criterion (AIC) value. Differences between conditions were evaluated using Tukey-adjusted least-squared means with a 95% confidence interval (CI). Lastly, we assumed no carryover effect would be observed given the nature of the intervention, the fact the intervention was only once-weekly, and that a two-week washout period was employed between treatment conditions. No formal sample size and power calculations were undertaken given this was a pilot study, with the objective of assessing intervention trends in outcome measures and feasibility. Therefore, a total analytical sample size of 23 was considered adequate as suggested in previous literature [45].

## 3. Results

**Figure 2:**
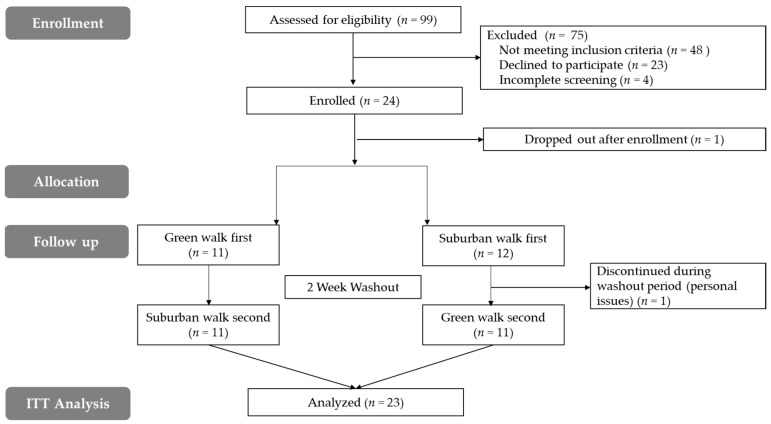
CONSORT flow diagram—modified for non-randomized crossover trial design.

Figure 2 shows a modified CONSORT participant flow diagram. One participant was excluded from analyses because he/she dropped out after signing the consent form and therefore did not contribute any outcomes data to the study. A second participant discontinued during the washout period for reasons unrelated to the study. This participant was included in the analyses per intention-to-treat (ITT) protocol suggested by the CONSORT guidelines [46]. Demographic characteristics from these participants were similar to study completers. 

Table 1 presents participants’ selected baseline demographic and anthropometric characteristics. On average, the sample was comprised mostly of middle-aged females, who were obese and had college or some college-level education. Table 2 presents pre- and post-walk mean scores for the psychological questionnaires and visual BDS task, in addition to the between condition contrast at the post-walk assessment. 

Anxiety. Pre-walk mean anxiety scores were similar between conditions (*p* = 0.6). Results revealed lower mean anxiety scores post-green walking sessions compared to post-suburban walking sessions (−2.5, 95% CI (−4.5, −0.5); *p* = 0.02). 

Mood. Pre-walk mean positive affect and negative affect scores were similar between conditions (*p* = 0.5 and *p* = 0.6, respectively). Mean positive affect scores were higher post-green walking sessions relative to post-suburban walking sessions (2.0, 95% CI (0.2, 3.9); *p* = 0.03). Further, although mean negative affect scores were somewhat lower post-green walking sessions relative to post-suburban walking sessions, the post-walk mean negative affect scores remained similar between conditions (−0.7, 95% CI (−1.4, 0.04); *p* = 0.07). 

Directed-attention. Pre-walk mean directed-attention scores were similar between conditions (*p* = 0.6). After adjustment for a potential learned effect, results indicated similar directed-attention scores for post-walk sessions between the green and suburban walking conditions (−0.1, 95% CI (−0.8, 0.5); *p* = 0.6).

## 4. Discussion

This study’s purpose was to examine acute changes pre- to post-walk for anxiety, mood, and directed-attention between repeated walking sessions in a green environment compared to a suburban environment. Results suggested that green walking reduced state anxiety and improved positive affect better than suburban walking, with green walking eliciting somewhat greater reductions in negative affect versus suburban walking. Directed-attention did not seem to benefit from either green walking or suburban walking in this sample. 

Consistent with our first hypothesis, greater reductions in state anxiety were observed after green walking versus suburban walking. Further, the larger improvements in positive and negative affect seen after green walking relative to suburban walking were aligned with our second hypothesis. While the psychological benefits of PA, including improvements in overall mental health, well-being, and cognitive abilities [47,48,49] are better understood, previous studies have hypothesized that additional psychological benefits might be gained when performing green exercise as green environments may have restorative effects capable of eliciting greater anxiety reductions and mood improvements than environments with lesser degrees of greenspace [50]. Among studies which have investigated the effect of green walking on anxiety and mood, these studies have largely indicated improvements in aforementioned and hypothesized directions [51,52,53,54,55,56]—consistent with this study’s observations. In fact, past studies demonstrated that greater improvements on anxiety and/or mood can be promoted by simply exposing participants to views of nature [16,17,57] relative to exposure to urban views. Although the comparison groups for several of these studies included urban environments as opposed to suburban environments like that of our study, our observations contribute to this literature by comparing how these outcomes differed between a green environment and an environment within which an individual might walk more frequently for exercise (e.g., on a suburban sidewalk near their residence). Specifically, the current study suggested that regular green walking may have greater beneficial effects on psychological outcomes (e.g., anxiety and mood) beyond that observed when completing regular walks in suburban environments.

As a majority (52%) of Americans live in suburban areas [58], future well-powered studies are encouraged to compare the psychological benefits of regular green walking relative to suburban walking, with the objective being to discern what real-world land use policy changes might benefit the health of these communities. For example, these studies could seek to provide evidence of how improving the access of suburban communities to recreational parks or forests improves psychological (and physiological) health. Further study is also warranted to understand what green environment characteristics (e.g., less air and noise pollution) most improve acute anxiety and mood indices after walking exercise, and how these characteristics may also maximize well-established physiological health benefits of PA, like reduced blood pressure, improved weight maintenance, and cardiovascular disease prevention [39].

Greater improvements in attention during green walking compared to suburban walking were not observed—unsupportive of our final hypothesis. It is noteworthy, however, that these observations are aligned with other studies that did not find green walking to elicit greater attention restoration relative to walking in an urban environment. Specifically, Hartig and colleagues [51] reported that walking in green environments did not improve post-walk memory task performance compared to walking in an urban environment. Similarly, other studies reported no performance improvements pre- to post-walk on the digit span test after nature walks compared to walks in an urban environment [9,59,60]. Conversely, Berman et al. [17] reported that green walking improved attention (i.e., had a restorative effect on attentional capacity) relative to walking in urban environments. A potential explanation as to why our observations and most other literature differed from that of Berman and colleagues [17] may be due to the fact participants in these researchers’ study were exposed not only to the BDS task, but also to a fatigue-inducing task of approximately 35 min in duration prior to the walk. These researchers hypothesized that exposure to the fatigue-inducing task might have increased participants’ sensitivity to the restorative effects of the green environment during the walk [17]. Given these inconsistent findings, more research is needed to elucidate if exposure to, or exercise within green environments would elicit greater potential for attention restoration relative to suburban (or urban) environments.

This pilot study has strengths and limitations. The main strengths include: (1) the use of a crossover design with proper washout period, which increases statistical precision and power among smaller samples, and reduces the chances of carryover effects, respectively; and (2) implementation of a PA modality completed at intensity and frequency easily achieved by most individuals regardless of physical fitness—thereby increasing study observations’ generalizability. However, several limitations to this study warrant specific mention. First, due to resource limitations, simultaneous random assignment to treatment sequence was not possible. Although this could make the study susceptible to confounding factors that could lead to biased estimates, this is less of a concern for studies employing crossover designs because each participant serves as his/her own control—which increases statistical precision when estimating treatment effects [32]. Second, anxiety, mood, and directed-attention were all measured using self-report questionnaires, with no proxy physiological measures of these outcomes reported concurrently. Third, following study completion, we noticed that one of the 4-point Likert-type scale responses for the STAI state questionnaire was inadvertently listed as “frequently so” instead of the original “very much so”. While we do not believe this to be the case, this unintentional mistake could have led to measurement error. Fourth, although we do not believe that participants interacted with others during these walks, we cannot rule out these behaviors. However, it is worth noting that there was low foot traffic on the trails/sidewalks during the weekdays on which the walks were conducted and, further, that we staggered participant start times to ensure all walks were completed alone. Fifth, although promising, these preliminary results should be interpreted in the context of a pilot study and need to be further examined in larger experimental studies. Finally, this pilot study used a homogenous convenience sample —comprised of mostly college-educated healthy women of middle- to high-income from a single geographic area. Thus, this study has limited generalizability. Future studies should aim to recruit more diverse and high-risk participants, and from multiple geographic locations. 

## 5. Conclusions

This pilot study assessed the acute effects of regular walking sessions in different outdoor environments (i.e., green and suburban) on psychological and cognitive outcomes using a crossover design in a sample of adults. Study observations contribute to current green exercise research by demonstrating the potentially greater beneficial effects of repeated green walking sessions on state anxiety and positive affect compared to walking sessions completed in more suburban environments. However, observations did not convincingly suggest that green walking promoted greater beneficial outcomes for attention restoration relative to suburban walking. Nonetheless, future larger, more generalizable, and methodologically rigorous studies comparing the differential acute and chronic effects of green walking to that of walking in suburban or urban areas are warranted to continue addressing the noted literature gaps [7,38] and calls for greenspace conservation [10]. Indeed, if methodologically rigorous studies of green walking can continue to compellingly demonstrate that PA in green environments can promote greater psychological wellness and simultaneously contribute to improved physiological health, regular green walking might be more frequently prescribed by health professionals for adults as an alternative/holistic approach for the prevention and treatment of diseases and to improve health outcomes. 

## Figures and Tables

**Table 1:** Selected baseline demographic and anthropometric characteristics of participants included in the analytical sample.

Characteristics	All (*n* = 23)
Age, mean ± SD, year	49.7 ± 6.5
Female, *n* (%)	19 (83)
Education, *n* (%)	
College/Some college	17 (74)
Graduate level	6 (26)
Income, n (%) *	
<49,000	4 (19)
50,000–99,999	8 (38)
100,000 or more	9 (43)
Exercise, mean ± SD, days/week	1.7 ± 0.6
BMI, mean ± SD, kg/m^2^	31.0 ± 7.9
Body fat percentage, mean ± SD	38.4 (10.1)

Abbreviations: Physical activity, PA; body mass index, BMI; kilograms/meter squared, kg/m^2^. * Missing data were not considered in determining percentages (two missing).

**Table 2:** Pre- and post-walk mean scores, and post-walk contrasts between-treatment conditions for psychological and cognitive outcome measures by green and suburban conditions (*n* = 23).

Outcome Measures	Green		Suburban		Between Condition Contrast
	Pre-Walk	Post-Walk	Pre-Walk	Post-Walk	
	*mean* ± SE		*mean* ± SE		*mean* (95% CI)
STAI-S ^a^	30.0 ± 1.4	28.2 ± 1.6	29.5 ± 1.4	30.6 ± 1.6	−2.5 (−4.5, −0.5)
PANAS ^b^					
Positive Affect	35.3 ± 1.5	37.6 ± 1.6 *	35.9 ± 1.5	35.6 ± 1.5	2.0 (0.2, 3.9)
Negative Affect	11.5 ± 0.6	11.0 ± 0.4 ^†^	11.7 ± 0.6	11.7 ± 0.4	−0.7 (−1.4, 0.04)
BDS ^c^	6.5 ± 0.4	6.4 ± 0.4	6.6 ± 0.4	6.5 ± 0.4	−0.1 (−0.8, 0.5)

Notes. ^a^ STAI-S (anxiety): range 10–40, higher scores = greater anxiety; ^b^ PANAS (Positive Affect and Negative Affect): range 10–50, higher scores on positive and negative affect = higher positive or negative emotional states, respectively; ^c^ BDS (directed-attention): range 0–14, higher scores = higher directed-attention; within-condition, pre- to post-walk mean score changes: * *p* = 0.003, ^†^ *p* = 0.04.
